# THE FORMULA FOR CARDIOVASCULAR HEALTH AND WELL-BEING IN ADULTS WITH CEREBRAL PALSY

**DOI:** 10.1093/geroni/igac059.517

**Published:** 2022-12-20

**Authors:** Patrick McPhee

**Affiliations:** McMaster University, Hamilton, Ontario, Canada

## Abstract

Persons with cerebral palsy (CP) have mobility limitations, heightened sedentary behavior, and are at increased risk for cardiovascular disease (CVD). Dr. McPhee will cover his innovative work to develop a core outcome set for cardiometabolic disease risk assessment in adults with CP, and will further discuss recent findings from ongoing work pertaining to novel CVD risk indicators showing accelerating cardiovascular aging in CP. Dr. McPhee will also present recent work on the role of exercise, sleep, and healthy nutrition in the context of healthy aging for individuals with CP across the lifespan.

